# Regulatory T Cells and Ocular Graft Versus Host Disease: A Novel Treatment Approach

**Published:** 2018

**Authors:** Mohammad Reza Pishnamaz, Ebrahim Jafarzadehpour, Razieh Pishnamaz

**Affiliations:** 1 Optometry Department, Iran University of Medical Sciences, Tehran, Iran; 2 Department of Allergy and Immunology, Mashhad University of Medical Sciences, Mashhad, Iran

**Keywords:** Graft vs Host Disease, T-Lymphocytes, Regulatory, Anti-IL-27p28–Specific Antibody, IL-27 Receptor

## Abstract

Graft Versus Host Disease (GVHD) is an inflammatory immune disease, mediated by the donor’s immune cells and can arise after allogeneic Hematopoietic Stem Cell Transplantation (HSCT) for the treatment of hematologic malignancies. It can lead to destructive manifestations in various tissues, particularly dermatological, gastrointestinal, and ocular tissues. The most common ocular morbidity is dry eyes, which is often the first manifestation of GVHD. Regulatory T cells (Tr) can be broadly classified as natural or adaptive (induced). After Bone-Marrow Transplantation (BMT), excessively increased levels of type 1 Tr (Tr1) are generally observed with absence of a GVHD, while low levels are seen with severe GVHD. Treatment of patients, undergoing BMT with Interleukin-10 (IL-10)-anergized donor T cells, led to immune reconstitution without the development of GVHD, which resulted in protection against infection and against the return of the cancer. Surprisingly, in both naive syngeneic mouse models of skin and cardiac allografts, graft retention was augmented after infusion of in vitro generated double-negative Tr (DN Tr). In addition, GVHD was reduced in mice with a genetic deficiency in the IL-27 receptor (IL-27R-/-) and in mice treated with anti-IL-27p28–specific antibody. Considering above mentioned findings we would suggest carrying out experiments, using animal models of GVHD, in order to evaluate the potential role of Tr, as an innovative approach to overcome severe ocular morbidity caused by ocular GVHD.

## INTRODUCTION

Graft Versus Host Disease (GVHD) is an inflammatory immune disease mediated by the donor’s immune cells, and can lead to the destruction of various host tissues [1]. Surprisingly, genetic dissimilarities between host and donor has proved to be ineffective for identification of auto-epitopes in this immune disease [2]. It can manifest either as acute or chronic GVHD (aGVHD and cGVHD, respectively), which are differentiated from each other according to their clinical manifestations. In 12% to 17% of patients with aGVHD, ocular manifestations occur, such as acute hemorrhagic conjunctivitis and pseudomembranous conjunctivitis. Exceedingly complex immunopathological mechanisms have been recognized for clinical features of cGVHD, and role of donor B and T cells besides other immune effector cells has been proven [3-5]. The GVHD can arise after allogeneic Hematopoietic Stem Cell Transplantation (HSCT) for the treatment of hematologic malignancies. This occurs because of a reaction against allo-antigens on the surface of the recipient’s cells, similar to the beneficial graft-versus-tumor reaction, initiated by the donor’s immune cells against cancer cells [6, 7]. Once initiated, it can lead to destructive manifestations in various tissues, particularly dermatological, gastrointestinal, and ocular tissues. The most common ocular morbidity is dry eyes, which is often the first manifestation of GVHD. However, severe forms of GVHD can be lethal [4, 8].

Following the discovery of regulatory T cells (Tr), in 1995, as a subpopulation of CD25^+^CD4^+^T cells, immunologists suspected that these effector T cells had a suppressive role due to their expression of CD25. In 2003, the Forkhead Box P3 (FOXP3) transcription factor was identified as an essential marker of a subset of Tr that played a suppressive role in the immune system [9]. In general, Tr can be broadly classified as natural or adaptive (induced). Both of these cell types are responsible for preserving self-tolerance and preventing excessive immune responses against foreign antigens. After Bone-Marrow Transplantation (BMT), excessively increased levels of type 1 Tr (Tr1) are generally observed with absence of aGVHD, while low levels are seen with severe GVHD. Therefore, a growing number of trials have been investigating the potential role of Tr1 for both treating and preventing GVHD after BMT. To achieve this goal, treatment of patients undergoing BMT with Interleukin 10 (IL-10)-anergized donor T cells has been explored [10]. This treatment has been found to lead to immune reconstitution without the development of GVHD, which resulted in protection against infection and against the return of cancer [10].

In rodents, there is a special type of Tr, CD4^-^CD8^-^CD3^+^Tr, which is known as double-negative Tr (DN Tr). These cells exhibit unique surface markers, including CD69, CD45, CD30, CD62L, CD25, lymphocyte function-associated antigen 1 (LFA-1), T-cell receptor alpha/beta (TCRαβ), and cytotoxic T-lymphocyte-associated protein 4 (CTLA-4). Once activated, the cells can produce Tumor Necrosis Factor-alpha (TNF-α), Interferon-gamma (INF-γ), and Transforming Growth Factor-beta (TGF-β). In vitro and in vivo investigations have revealed the suppressive role of DN Tr on CD8^+^ and CD4^+^ T cell responses. Surprisingly, in both naive syngeneic mouse models of skin and cardiac allografts, graft retention was augmented after infusion of in vitro-generated DN Tr [11, 12].

In another animal study, GVHD was reduced in mice with a genetic deficiency in the IL-27 receptor (IL-27R^-/-^) and in mice treated with anti-IL-27p28–specific antibody. Further investigations revealed that shifting the donor T-cell immune response away from pathogenic Tbet^+^CD4^+^type 1 T-helper cells and CD8^+^type 1 cytotoxic T cells, and towards CD4^+^ and CD8^+^ FOXP3-expressing Tr, significantly reduces GVHD grading in mice. In addition, an in vivo IL-27 blockade did not adversely affect IL-10 production by Tr and enhanced the stability of these cells during GVHD in mice [13]. Considering its evident efficacy in vitro and in animal models, a growing number of in vivo studies are currently applying Tr as a cell therapy in GVHD [14, 15].

## IMPLICATIONS

Considering the above-mentioned findings, the current authors recommend carrying out experiments, using animal models of GVHD, in order to evaluate the potential role of Tr, as an innovative approach to overcome severe ocular morbidity caused by ocular GVHD.
